# CounterAKTing HIV: Toward a “Block and Clear” Strategy?

**DOI:** 10.3389/fcimb.2022.827717

**Published:** 2022-02-04

**Authors:** Sébastien Pasquereau, Georges Herbein

**Affiliations:** ^1^Laboratory Pathogens & Inflammation-Epigenetics of Viral Infections and Inflammatory Diseases Laboratory (EPILAB), University of Franche-Comté, Bourgogne Franche-Comté University Bourgogne Franche-Comté (UBFC), Besançon, France; ^2^Laboratory of Virology, Centre Hospitalier Universitaire (CHU) Besançon University Hospital, Besançon, France

**Keywords:** HIV, Akt, PI3K, latency, reactivation, shock-and-kill, block-and-lock, block-and-clear

## Abstract

The protein kinase B or Akt is a central regulator of survival, metabolism, growth and proliferation of the cells and is known to be targeted by various viral pathogens, including HIV-1. The central role of Akt makes it a critical player in HIV-1 pathogenesis, notably by affecting viral entry, latency and reactivation, cell survival, viral spread and immune response to the infection. Several HIV proteins activate the PI3K/Akt pathway, to fuel the progression of the infection. Targeting Akt could help control HIV-1 entry, viral latency/replication, cell survival of infected cells, HIV spread from cell-to-cell, and the immune microenvironment which could ultimately allow to curtail the size of the HIV reservoir. Beside the “shock and kill” and “block and lock” strategies, the use of Akt inhibitors in combination with latency inducing agents, could favor the clearance of infected cells and be part of new therapeutic strategies with the goal to “block and clear” HIV.

## Introduction

The protein kinase B or Akt is a central regulator of survival, metabolism, growth and proliferation of the cells (Malim and Emerman, 2008) and is known to be targeted by various viral pathogens, including HIV-1 (Diehl and Schaal, 2013). The phosphoinositide 3-kinase (PI3K) is the effector upstream of Akt and is responsible for the signal transduction that follows the activation of some transmembrane receptors (So and Fruman, 2012). Several isoforms, notably class I isoforms α, β, γ and δ, have been identified and differentially associated with the regulation of metabolism, angiogenesis, or immunity (Bilanges et al., 2019). As such, it is a key molecule in the B and T cells development, activation and differentiation. The mammalian target of rapamycin (mTOR) is a downstream effector of Akt and it is involved in both T cells and B cells activation and differentiation (Limon and Fruman, 2012; Waickman and Powell, 2012). Thus, the PI3K/Akt pathway is involved in several key cellular processes that are involved in the progression of HIV-1 pathogenesis (Fayard et al., 2010). HIV infection is characterized by a latency phase and the presence of long-lived cellular reservoirs, from which viral reactivation occurs. The establishment of these viral reservoirs requires the regulation of cellular pathways activation to induce transcriptional silencing of the viral genome, and the enhancement of cell survival, notably by reducing the stress-induced apoptosis. Akt is involved in the regulation of cell survival, notably in response to viral entry, to enhance the survival of infected cells, allowing the establishment of viral reservoirs. Akt is also involved in the regulation of the HIV-1 transcription by regulating several cellular factors, including the transcription factor NF-kB. The Akt pathway has also been shown to be a key host factor for HIV replication (Zhou et al., 2008). Given its predominant cellular and viral regulatory function, the knowledge of HIV interactions with the PI3K/Akt pathway could help target key points in the pathogenesis progression to counter it.

## Critical Role for Akt in HIV-1 Pathogenesis

### PI3K/Akt Pathway Activation Favors Virus Entry Through Cofilin

**Figure 1:**
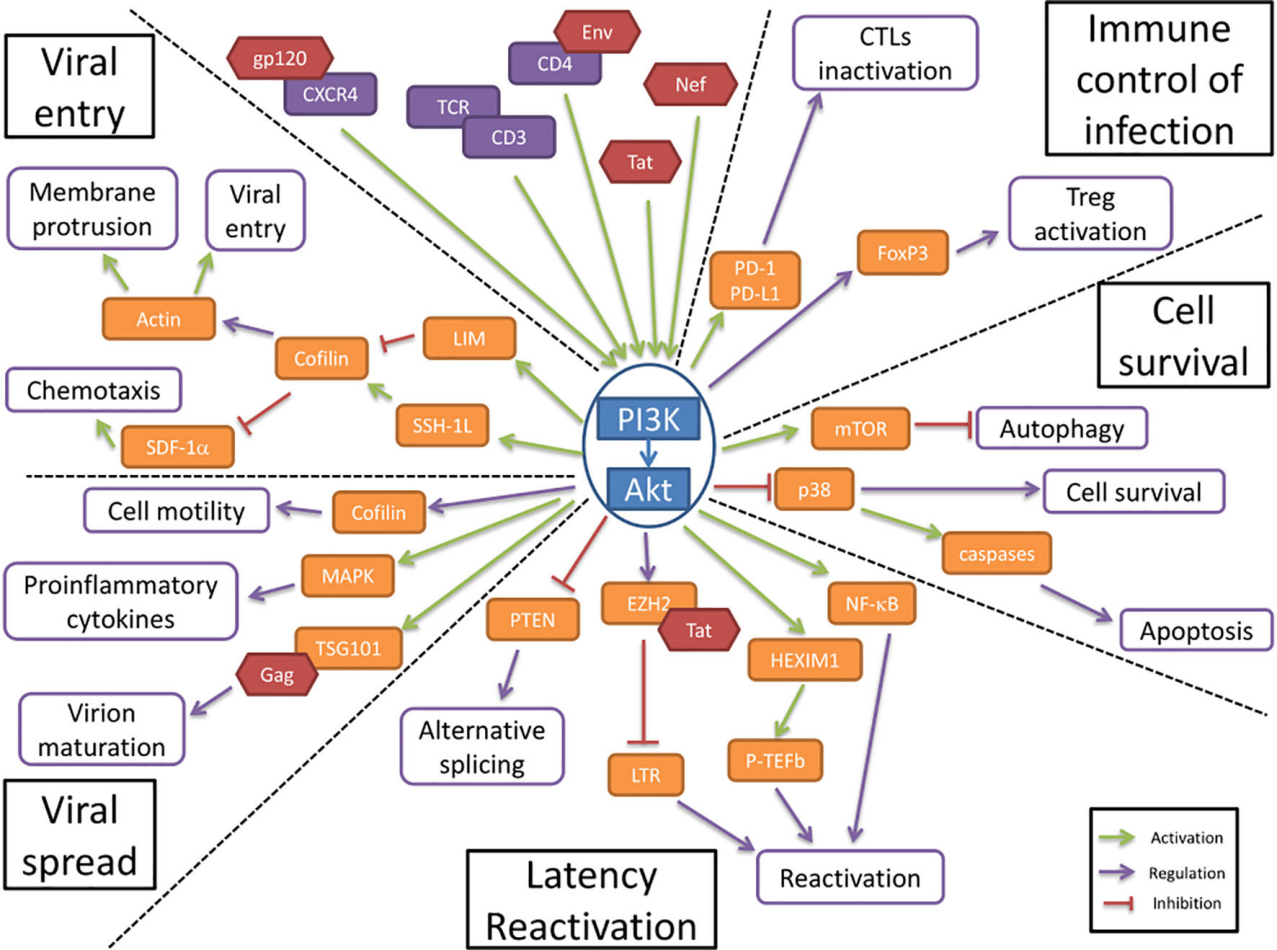
The PI3K/AKT pathway is involved in HIV-1 pathogenesis. The PI3K/Akt pathway plays a central role in HIV-1 pathogenesis by regulating several molecular axes both at cellular and viral levels. The PI3K/Akt pathway is activated by several viral proteins. It is involved in the regulation of the immune control of infection and cell survival. The activation of the PI3K/Akt pathway by viral proteins induces an increase in cell survival and an inhibition of the immune response to the infection. The PI3K/Akt axis interferes with HIV latency and reactivation, favors viral entry and participates in the increased viral spread.

The first step in HIV pathogenesis is the virus entry into the cells, for which Akt regulatory functions are altered. Static cortical actin is restricting HIV entry in T cells, and this restriction is lifted through the activation of cofilin. Cofilin activation has been shown to enhance the latent infection of resting T cells, and actin activity appears to be crucial for HIV-1 latent infection of resting T cells (Wang et al., 2012). The PI3K/Akt pathway activates cofilin through the phosphatase Slingshot-1L (SSH-1L), favoring plasma membrane protrusion and viral entry (Nishita et al., 2004)(**Figure 1**). HIV-1 gp120 protein binding to CXCR4 induces PI3K activity and LIM domain kinase transient activation, which will in turn deactivate cofilin, inducing actin polymerization (François and Klotman, 2003; Vorster et al., 2011). PI3K activation by gp120 will result in the downstream activation of two antagonist pathways (LIM and SSH) that will regulate cofilin activity. The impact of cofilin and actin remodeling on the viral entry was shown in the process of spinoculation, widely used to enhance *in vitro* viral infection. The increase in cofilin activity triggered by the spin promotes cytoskeletal dynamics that increase viral entry (Guo et al., 2011). The contribution of cofilin activation to HIV infection has been observed in clinical settings too, where infected patients presented higher levels of active cofilin (Wu et al., 2008). gp120 has also been shown to inhibit SDF-1α-induced chemotaxis. This inhibition appears to be mediated by cofilin activation (Trushin et al., 2010). Another viral protein is implicated in the modulation of cell motility and cytoskeleton remodeling. HIV-1 Nef is a key factor that induces cofilin inactivation, leading to a dysregulation of the actin remodeling in T cells (Stolp et al., 2009). Nef has been shown to bind to PI3K and Akt, contributing to the hijacking of cellular regulatory pathways by the HIV, to favor the infection (Wolf et al., 2001; Kumar et al., 2016b).

### Akt Favors HIV-1 Latency and Reactivation

In resting CD4+ T cells, HIV-1 latency can be established without cell activation. The nuclear localization and integration of HIV-1 is associated with exposure to various chemokines and the rapid activation of cofilin. These processes both involve the Akt pathway (Cameron et al., 2010). HIV-1 protein Nef activates the PI3K/Akt pathway and downregulates the inhibitor protein PTEN. This Akt activation, coupled with the induction of miR-718, results in the alteration of the phosphorylation pattern of serine rich proteins involved in the regulation of translation. An alternative splicing pattern of HIV-1 mRNAs is favored as a result of this dysregulation, leading to a reduction of viral replication (Diehl and Schaal, 2013). The PI3K/Akt pathway modulation of splicing seems essential for the establishment of latency (**Figure 1**).

HIV-1 reactivation is mediated by the positive transcription elongation factor b (P-TEFb), which is under control of regulatory protein complexes, including a HEXIM1/7SK snRNA complex and CTIP2 (Cherrier et al., 2013). P-TEFb expression has been shown to be under the control of both PI3K/Akt and ERK1/2 pathways for the reactivation of latent HIV (Mbonye et al., 2021). The phosphorylation of HEXIMI1 induces the release of transcription inhibition and is subsequent to the activation of the PI3K/Akt pathway (Contreras et al., 2007). The transactivation of the long terminal repeats (LTR) of the HIV-1 genome is also triggered by HIV-1 proteins, namely Tat. This activation is mediated through the PI3K/Akt pathway, alongside p65 and IKK phosphorylation (Zhang et al., 2011a). Tat additionally stabilizes Mdm2 through its phosphorylation by Akt, which enhances the transactivation of the LTR (Raja et al., 2017). Tat interacts with other regulatory proteins of the LTR activity, including EZH2. EZH2 inhibition of the transcription is lifted by its phosphorylation which is mediated by Akt (Zhang et al., 2015). The viral protein Nef induces the activation of T cells, and therefore activates the production of cytokines, including IL-2. This effect is mediated through the activation of NF-κB as a result of the direct interaction between Nef and Akt (Kumar et al., 2016a). HIV-1 reactivation is also induced by extracellular vesicles, notably exosomes, from uninfected cells, regardless of treatment. This extracellular activation is driven *via* the PI3K/Akt pathway (Barclay et al., 2020)

### Akt Activation Enhances Cell Survival

The HIV Envelope (Env) glycoprotein binding to CD4 cell surface receptors induces Akt activation, suppressing p38 MAP kinase activation, promoting cell survival (Li and Pauza, 2013) (**Figure 1**). It also increases the expression of PD-1, Fas and FasL by binding to CCR5 receptors, inducing cell death. This increase in apoptosis is countered by Akt activation, which reduces the p38 activation of caspases. The viral protein Nef induces the activation of PI3K directly at the plasma membrane, which in turn targets the pro-apoptotic factor Bad, blocking apoptosis in T cells (Wolf et al., 2001). In addition, the viral protein Tat also enhances Akt activation, resulting in an anti-apoptotic effect (Borgatti et al., 1997).

Autophagy appears to be an important target in HIV infection, mainly during viral replication. mTOR, an effector downstream of Akt, regulates autophagy and could be targeted by HIV-1 (Dinkins et al., 2015). In dendritic cells, the Env viral protein induces mTOR activation, and in macrophages Nef has been shown to inhibit autophagy by binding to Beclin-1 to possibly increase mTOR activation (Campbell et al., 2015). The activation of mTOR by HIV-1 appears to be associated with PI3K/Akt activation. The hijacking of the PI3K/Akt/mTOR pathway by HIV-1 enhances the cell survival by inhibition of autophagy (Le Sage et al., 2016). Autophagy activation upon viral infection is responsible for an important anti-HIV effect, by the targeted degradation of Tat, thus inhibiting HIV replication (Sagnier et al., 2014). The inhibition of autophagy at later stages, notably through the PI3K/Akt activation, could be an important player in the reactivation from latency (Daussy et al., 2015).

### Akt Favors Viral Spread

Cellular adherence has been shown to induce protection from apoptosis, by activation of PI3K/Akt. This cell surface contact induced activation of PI3K/Akt pathway is also present in cell-to-cell contact in T cells, which is the main mode of viral transmission *in vivo* (Watton and Downward, 1999; Titanji et al., 2013). Akt activation, by regulating cell motility, could favor the viral spread by cell-to-cell contact (**Figure 1**). It is important to note that cell motility is tightly linked to actin remodeling and therefore under the influence of cofilin, which is a key point in the pathogenesis, as described above (Stolp et al., 2009).

The HIV-1 envelope glycoprotein gp120 has been shown to stimulate the production of several cytokines, including TNF-alpha, in macrophages. This stimulation has been determined to be under the control of PI3K and the downstream kinase MAPK, as well as associated with p38 and ERK-1/2 (Lee et al., 2005). This proinflammatory cytokines production help stimulate bystander cells, increasing the size of the HIV reservoir (Pasquereau et al., 2018).

The endosomal sorting complex required for transport (ESCRT) is a cellular machinery associated with ubiquitination and is needed by the HIV to achieve virion maturation and release. The viral protein Gag directly binds to the ESCRT-I subunit TSG101, in order to undergo ubiquitination (Sette et al., 2013). TSG101 has been shown to directly interact, in a constitutive manner, with Akt, which induces an increase in viral budding and virion release. The disruption of this interaction is associated with the CXCR4-mediated Akt activation (Verma and Marchese, 2013).

### Akt Activation Decreases the Immune Control of Infection

The control of HIV infection by the immune system involves several cell populations, that are targeted by the virus to allow evasion.

CTL cells activation is regulated through the expression of PD-1 on the cell surface, where it will bind to its ligands, PD-L1/2, expressed on the surface of APCs. This interaction will induce the deactivation of CTLs. In infected cells, HIV-1 induces the increase of PD-1 expression in CTLs, as well as the expression of PD-L1 in APCs. This ligand upregulation is under the influence of the PI3K/Akt pathway activation, by both transcriptional and post-translational mechanisms (Muthumani et al., 2011; Chen et al., 2016) (**Figure 1**). In turn, the PD-1 engagement induces the inhibition of Akt activation, mediated by TCR dephosphorylation, suggesting a regulatory role in the maintenance of latency reservoir (Kulpa et al., 2013).

Regulatory T cells (Tregs) activation is associated with increased Foxp3 expression, which appears to determine the differentiation between effector or regulator cells. The Foxp3 expression in CD4 T cells following TCR triggering is regulated by the PI3K/Akt pathway (Etemire et al., 2013). The constitutive PI3K activity that results from TCR signaling will result in the overexpression of Foxp3, leading to an increase in regulatory T cells activation (Sauer et al., 2008). This regulatory T cells activation during acute infection help establish a high level of HIV-1 replication, by modulating the anti-HIV immune response. During latency however, the lower activation of Tregs participates in the establishment of the viral reservoirs (Holmes et al., 2008).

It has been demonstrated *ex vivo* that NK cells inhibit HIV-1 replication in both T cells and macrophages, suggesting an important regulatory role in the HIV-1 infection *in vivo* (Fehniger et al., 1998). NK cells activation is modulated by numerous cytokines, including interleukin-15 (IL-15). The IL-15 response of NK cells is mediated by the PI3K/Akt pathway (Ali et al., 2015). Studies have shown that, during the acute HIV infection, IL-15 is produced and affects the viremia, by acting not only on NK cells, but on T cells too (Mueller and Katsikis, 2010).

In the central nervous system, HIV-1 induces a dysregulation of inflammation in astrocytes and microglia, responsible for HIV-associated neurocognitive disorders (HAND) (Hong and Banks, 2015). HIV-1 Tat notably induces pro-inflammatory cytokines production, *via* several signaling pathways including PI3K/Akt (Zhou et al., 2019).

### AKT Activation Favors Macrophage Survival and HIV-1 Replication

In macrophages, there appears to be a dynamic role for Akt activation during the viral life cycle. In the early infection stages, Akt activation induces FOXO3a inactivation, leading to cell death resistance (Cui et al., 2008; Zhang et al., 2011b). This allows for viral replication and accumulation within the cell. This increase viral replication will in turn induce the downregulation of the PI3K/Akt pathway, therefore lifting the restriction on FOXO3a which is involved with other factors such as TRAIL or Fas in cell death (Cui et al., 2009). The extended life span of HIV-1 infected macrophages, mediated by the PI3K/Akt activation, make them important viral reservoir during HIV latency. The activation of Akt in macrophages regulates macrophages activation, by downregulating the proinflammatory responses to induce a polarization shift towards anti-inflammatory responses (Vergadi et al., 2017). This shift in macrophage activation could participate in the establishment of viral latency.

## Akt Targeting as a Therapy to Counter HIV Infection

Due to its critical role in HIV pathogenesis, targeting PI3K/Akt pathway could control HIV-1 entry, viral latency/replication, cell survival of infected cells, HIV spread from cell-to-cell, and the immune microenvironment which could ultimately allow to curtail the size of the HIV reservoir. PI3K and Akt inhibitors have been developed, notably as anticancer drugs, and these drugs could be repurposed to counter HIV infection. A list of inhibitors can be found in **Table 1**. Extensive reviews of these drugs, including their targets and development status, have been published by others (Wang et al., 2015; Martorana et al., 2021).

**Table 1:** PI3K and Akt inhibitors currently available or under development.

Class	Name	Target	Use	Status
PI3K inhibitors	Alpelisib	Class I PI3K	Anticancer drug	Available
	Serabelisib	Class I PI3K	Anticancer drug	Phase II (NCT04073680)
	A66	Class I PI3K	Anticancer drug	Preclinical
	GSK2636771	PI3Kβ	Anticancer drug	Phase II (NCT04439188 and NCT04439149)
	AZD8186	Class I PI3K	Anticancer drug	Phase II (NCT04001569)
	AZD6482	Class I PI3K	Antiplatelet effect	Phase I (NCT00688714)
	Idelalisib	PI3Kα and PI3Kβ	Anticancer drug	Phase II (NCT03133221 and NCT02135133)
	Acalisib	Class I PI3K	Anticancer drug	Phase I (NCT01705847)
	IC-87114	Class I PI3K	Anticancer drug	Preclinical
	Copanlisib	Class I PI3K	Anticancer drug	Available
	Taselisib	Class I PI3K	Anticancer drug	Discontinued after Phase III
	Duvelisib	PI3Kδ and PI3Kγ	Anticancer drug	Available
	SAR405	Class I PI3K	Autophagy inhibitor	Preclinical
Akt inhibitors	Miransertib	Akt1/2/3	PROS and Proteus syndrome	Phase II (NCT04316546 and NCT04980872)
	BAY1125976	Akt1/2	Anticancer drug	Phase I (NCT01915576)
	MK-2206	Akt1/2/3	Anticancer drug	Phase II (NCT01258998, NCT01277757, NCT01307631, NCT01349933, NCT01283035 and NCT01604772)
	TAS 117	Akt1/2/3	Anticancer drug	Phase II (NCT03017521 and NCT04770246)
	Afuresertib	Akt1/2/3	Anticancer drug	Phase II (NCT04374630)
	Capivasertib	Akt1/2/3	Anticancer drug	Phase III (NCT04305496, NCT03997123, NCT04493853 and NCT04862663)
	Ipatasertib	Akt1/2/3	Anticancer drug	Phase III (NCT04650581, NCT04177108, NCT03072238, NCT03337724 and NCT04060862)
	Uprosertib	Akt1/2/3	Anticancer drug	Phase II (NCT01902173, NCT01989598 and NCT01979523)
	GSK690693	Akt1/2/3	Anticancer drug	Discontinued after Phase I

### Inhibition of HIV Entry and Preintegration Steps

Inhibitors of PI3K were shown to inhibit infection of CD4 T cells after integration occurred but prior to gene expression. The gp120-induced PI3K activity and downstream effectors activation can be block by specific PI3K inhibitors, as well as blocking antibodies directed against CCR5 and CXCR4 (François and Klotman, 2003). Inhibitors of PI3K were shown to block cell-to-cell fusion by gp120-CD4 interaction, specifically by inhibition of PI3K/PTEN pathway (Hamada et al., 2019). This could be used to inhibit the viral replication in T cells.

### Modulation of HIV-1 Reactivation/Latency

The hyperactivation of T cells favors the reactivation of HIV-1 from latently infected cells, through the activation of Akt. Contrary to the effect of Akt activation in resting T cells, the high levels of cellular activation and of Akt activation induce the overexpression and activation of transcription factors associated with HIV reactivation, notably NF-kB and P-TEFb. This Akt activation can be reduced by protease inhibitors (PI), leading to a limitation of HIV-1 reactivation from latently infected cells (Kumar et al., 2016a; Kumar et al., 2016b; Pasquereau et al., 2018). In addition, inhibition of the viral gene expression could also be achieved by targeting the Tat protein. An inhibitor of Tat transcriptional activity, BPRHIV001, has been shown to block viral gene expression, by acting on the p300 protein, a regulator of Tat function. This inhibitor induced a reduction in the phosphorylation of Akt, which is known to be associated with p300 protein stability (Lin et al., 2011). The association of Tat inhibitors with Akt targeting drugs could provide new therapeutic strategies to block the viral replication. mTOR activation, downstream of Akt, is associated with the reduction of autophagy. The use of PI3K inhibitors and mTOR inhibitors, such as rapamycin, is associated with an increase in autophagy, through the relocalization of TFEB (Campbell et al., 2015). Drugs targeting the mTOR pathway and the upstream PI3K/Akt pathway could be used to counter the HIV-1 induced inhibition of autophagy, and therefore alter the viral replication (Donia et al., 2010). Additionally, serum starvation has been show to induce HIV reactivation in *in vitro* models, and therapeutic approaches derived from it could complement the other strategies (Raja et al., 2018).

The proinflammatory cytokine production, including TNF-alpha and IL-6, is increased in HIV-1 infection, through NF-kB activation. The use of protease inhibitors has been shown to reduce NF-kB activation, as well as Akt activation, resulting in a decrease in proinflammatory cytokine production and a limit in HIV-1 transcription (Equils et al., 2004; Kumar et al., 2016b; Pasquereau et al., 2018). gp120-induced TNF-alpha production was shown to be blocked by PI3K inhibitors. This inhibition also induces a block of p28 and ERK1/2 activation and appears to be mediated by CCR5. This could allow the targeting of viral-induced proinflammatory cytokine production in macrophages, to slow HIV pathogenesis (Abbas et al., 2015; Pasquereau et al., 2018).

### Blockade of Cell Survival and Induction of Apoptosis

Proteases inhibitors were shown to inhibit caspases and proteasomes activity, resulting in an altered response to apoptosis stimuli (Pajonk et al., 2002; Badley, 2005). The reduction of Akt activation exhibited by PI treatment could also help in the reduction of anti-apoptotic signals induced by Nef and Tat. Direct inhibition of Nef induced Akt activation has been previously reported in T cells (Kumar et al., 2016b). Apoptosis-inducing treatments have been used for cancer treatment. These drugs, which includes Akt inhibitors, could help achieve viral clearance, by promoting apoptosis in infected cells, notably macrophages (Lucas et al., 2010). The induction of apoptosis could be mediated by TRAIL or by an increase in autophagy, both under the dependence of Akt activation (Huang et al., 2006; Campbell et al., 2020).

### Limitation of the Viral Spread and Modulation of the Immune Environment

Virion maturation is needed for the spread of infection, and has been shown to be associated with Akt activation. Protease inhibitors present an inhibitory effect on virion maturation, by blocking the enzymes responsible for the Gag-Pol polyprotein cleavage (Kaplan et al., 1993). This results in the block of maturation. Additionally, PI have been shown to reduce Akt activation, and therefore they could inhibit the subsequent ESCRT activation, which is required for maturation (Sette et al., 2013). Current classes of inhibitors used in cART were assessed for their potential to block cell-to-cell transfer of HIV-1. Protease inhibitors were found to be more potent than reverse transcriptase inhibitors to prevent cell-to-cell transfer in T cells. PIs were also found to be effective against cell-free diffusion, which is the other main mode of HIV-1 spread (Titanji et al., 2013).

NK cells implication in the regulation of HIV-1 replication is mediated by IL15. It has been shown *in vitro* that the use of an IL-15 superagonist could result in an increase effectiveness of the NK anti-HIV response in acute infection. NK cells activation *in vivo* could be an interesting new therapeutic goal to treat acute infection (Seay et al., 2015). Additionally, activated dendritic cells have been shown to improve the latent HIV purge resulting from TCR stimulation. The DCs activate the PI3K/Akt pathway in the targeted cells (van Montfort et al., 2019).

## Perspectives and Conclusion

**Figure 2:**
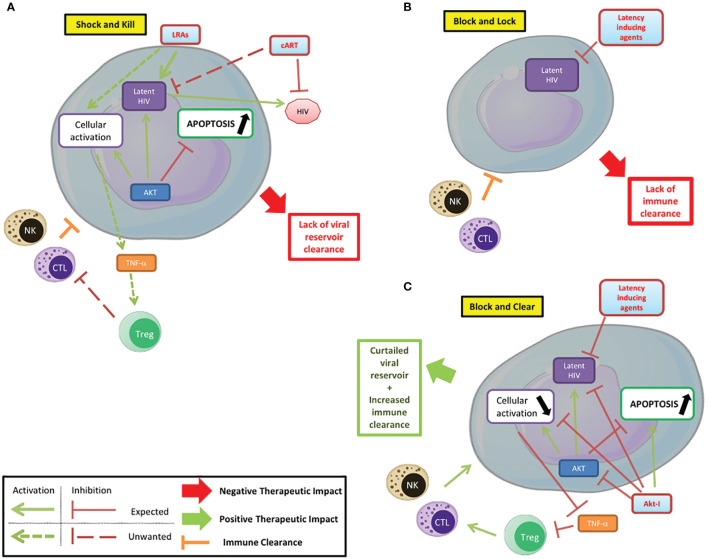
Targeting PI3K/AKT pathway to control HIV infection/reservoirs. **(A)** Shock and kill. The shock and kill strategy shows some limitations due to a lack of clearance of the HIV reservoir. **(B)** Block and lock. Latency inducing agents could prevent the reactivation from the latent reservoir, but the limited immune clearance of infected cells reduces the efficiency of this strategy based on the functional cure. **(C)** Block and clear. Beside the “shock and kill” and “block and lock” strategies, the use of Akt inhibitors in combination with latency inducing agents, could limit the cellular activation, favor the apoptosis of infected cells and their clearance and be part of new therapeutic strategies.

Akt plays a central role in cellular metabolism and particularly in cell survival. It is also a major player in HIV pathogenesis, participating in latency establishment and reactivation. Several steps of the HIV pathogenesis that involve Akt can be targeted by treatments. The use of PI3K/Akt inhibitors can help modulate the latency/reactivation of the virus and cell survival, as part of new therapeutic approaches and thereby complete the conventional “shock and kill” and “block and lock” strategies (**Figure 2**) (Herbein, 2020). In the “shock and kill” strategy, viral reactivation from latency is induced by latency reversing agents (LRAs) while infected cells are killed by cellular immunity. Known LRAs notably include PKC agonists, MAPK agonists, CCR5 agonists, HDACs inhibitors, HMT inhibitors and DNMT inhibitors (Ait-Ammar et al., 2020). To note, the Akt activator Disulfiram has been tested as an LRA. In the “block and lock” strategy, inhibition of viral reactivation prevents viral rebound by durable silencing of latent provirus. Both these strategies have been reviewed extensively, notably by Darcis et al. (Darcis et al., 2017). The use of Akt inhibitors could increase the clearance of infected cells. In fact, the pro-apoptotic effect of Akt inhibitors, as shown in cancer treatment could help clear the infected cells. However, this effect could be limited by the anti-reactivation effect of Akt inhibition if used in a “shock and kill” strategy. In addition, the different viral reservoirs are not impacted in the same way, with macrophages having a higher resistance to apoptosis than lymphocytes for example (Ait-Ammar et al., 2020). The penetration of latency reversing agents and Akt inhibitors into tissue reservoirs, especially in the central nervous system, is likely to be more limited (Wallet et al., 2019). The reduction in the viral reservoir induced by Akt inhibitors could be part of a “block and lock” strategy. Associated with reactivation inhibitors, notably Tat inhibitors, it could prevent any reactivation from the latent reservoir with the benefit of reducing the survival of infected cells by lifting the inhibition of apoptosis. This new paradigm could be named a “block and clear” strategy (**Figure 2C**). The inhibition of Akt could additionally reduce the cellular activation, notably in macrophages, further reducing the latent HIV reactivation and the viral spread to new reservoirs. PI3K/Akt inhibitors may also limit HIV entry into T-cells by blocking cofilin activation. Finally, the inhibition of the anti-apoptotic effects of Akt could help increase the clearance of infected cells by NK cells and CTLs. Overall, Akt is a major player of HIV pathogenesis, notably by its central regulatory role in lymphoid and myeloid cells. Targeting the PI3K/Akt pathway in the treatment of HIV could help overcome several problems with current therapeutic strategies that prevent the achievement of a functional cure in HIV infected patients. Beside the “shock and kill” and “block and lock” strategies, the use of Akt inhibitors in combination with latency inducing agents, could favor the clearance of infected cells and be part of new therapeutic strategies with the goal to “block and clear” HIV.

## Author Contributions

SP and GH wrote and edited the manuscript. All authors have approved of the manuscript for publication.

## Funding

This work was supported by grants from the University of Franche-Comté (UFC) (CR3300), the Région Franche-Comté (2021-Y-08292 and 2021-Y-08290) and the Ligue contre le Cancer (CR3304) to GH.

## Conflict of Interest

The authors declare that the research was conducted in the absence of any commercial or financial relationships that could be construed as a potential conflict of interest.

The editor declared a past co-authorship with one of the authors GH.

## Publisher’s Note

All claims expressed in this article are solely those of the authors and do not necessarily represent those of their affiliated organizations, or those of the publisher, the editors and the reviewers. Any product that may be evaluated in this article, or claim that may be made by its manufacturer, is not guaranteed or endorsed by the publisher.
